# The Influence of Titanium Dioxide Nanosheet on Water Permeability of Silicone Rubber after Nitrogen Dioxide Aging Treatment

**DOI:** 10.3390/ma16227138

**Published:** 2023-11-12

**Authors:** Xiangyang Peng, Jinshuai Zhang, Jiapeng Fang, Zheng Wang, Zhen Huang, Shilong Kuang, Chunqing He

**Affiliations:** 1 Guangdong Key Laboratory of Electric Power Equipment Reliability, Electric Power Research Institute of Guangdong Power Grid Co., Ltd., Guangzhou 510080, China; 2 Key Laboratory of Nuclear Solid State Physics Hubei Province, School of Physics and Technology, Wuhan University, Wuhan 430072, China

**Keywords:** water permeability, titanium dioxide nanosheet, silicone rubber, electrochemical performance

## Abstract

In this study, the aging process of a surface-functional titanium dioxide nanosheet (f-TNS) composited room-temperature-vulcanized silicone rubber (RTV) composite coating was simulated in a NO_2_ generation device, and then the electrochemical impedance spectroscopy (EIS) of the aged composite coating was tested in a 3.5% NaCl solution. The water permeation process was analyzed by the changes in the impedance modulus, porosity, and breakpoint frequency of the composite coating. The experimental results show that the water permeability of aged RTV decreases first and then increases with the increase in the composite proportion of f-TNS. When the composite proportion of TNS was 0.3 wt.%, the composite sample had the minimum water permeability and the best resistance to NO_2_ corrosion. The effect of TNS on the NO_2_ aging resistance of RTV composites and its mechanism were studied by SEM, FT-IR, and XPS. The impedance modulus and porosity of the aged 0.3% f-TNS/RTV, respectively, were 1.82 × 10^7^ Ω cm^2^ and 0.91 × 10^−4^%, which increased by 2.23 times and decreased by 0.37 times, respectively, compared with the values of aged pure RTV sample. In addition, the breakpoint frequency of the aged 0.3% f-TNS/RTV also significantly reduced to 11.3 Hz, whereas it was 35 Hz in aged pure RTV.

## 1. Introduction

Silicone rubber (SIR) materials [1] mainly contain high-temperature vulcanized SIR, addition-type fluid SIR, and room temperature vulcanized SIR (RTV) [2]. Because of their excellent thermal stability, mechanical strength, electrical insulation [3], and hydrophobicity characteristics, both are widely used in building decoration, medical cosmetology, daily necessities, and even aerospace and power facilities [4]. Composite insulators prepared by HTV and coated spray insulators prepared by RTV are used in the power industry on a large scale [5,6,7]. Composite insulators mainly use the Si−O and Si−CH_3_ chain structures of outer insulator SIR to bring about good hydrophobicity [8,9] and hydrophobic recovery [10], excellent anti-pollution flashover and electrical insulation properties, etc. At present, there are more than 10 million composite insulators in net operation [11]. However, since composite insulators always operate under the action of high-voltage electric field [12,13], in addition to the aging of common natural environmental factors such as light, thermal oxidation [14,15], popllution [16], and humidity [17,18], high-oxidizing NO_2_ produced by arc flashover and corona discharge caused by high voltage accelerate the decomposition of each component of SIR and increase the flaw in the body [19]. Studies have shown that the aging of NO_2_ can transform its basis Polydimethylsiloxane (PDMS) covered by SIR into hydrophilic and close inorganic silica. Firstly, PDMS network rupture and inorganic formation form nanopores, as well as a small number of micropores (Al(NO_3_)_3_) are generated near the surface, and then a large number of PDMS in the body layer produce a chain broken and become inorganic [20,21]. Some nano and micron holes for water diffusion are gradually formed and connected, causing the loss of hydrophobicity, anti-fouling flashover, and other characteristics, thus leading to the breakdown of internal insulation of composite insulators, broken series, and other malignant failures, posing a major threat to the operation of the power grid [22,23]. In other words, enhancement of the water permeability of SIR after NO_2_ aging has become an important bottleneck affecting its long-term application.

At present, various nanofillers, especially two-dimensional (2D) nanosheets, have extensive use in improving the gas barrier performance of polymer materials [24,25,26,27]. For example, Hara et al. mixed graphene oxide into a layered organosiloxane film to improve the water vapor barrier performance of the film, because the siloxane layer acts as the barrier layer for the diffusion of water molecules [28]. Zhang et al. found that when the packing content of g-C_3_N_4_ was 0.3 wt.%, the function g-C_3_N_4_/RTV had the best NO_2_ blocking performance [20]. Wen et al. used KH550 to modify the surface of 25 nm TiO_2_ nanoparticles to better disperse in a polyvinylidene fluoride (PVDF) matrix by the melt blending method. The lowest permeability coefficient of PVDF/0.5% N-TiO_2_ showed better mechanical and CO_2_-blocking properties [29]. Jia et al. mixed organic modified montmorillonite (OMMT) into SIR and found that the addition of OMMT significantly improved the N_2_ barrier performance of SIR [30]. Chen et al. assembled a Schottky junction-induced TiO_2_@Sn_3_O_4_ nano-heterojunction and found that it has a high sensitivity to NO_2_ gas sensing at room temperature [31]. Coincidentally, Liu et al. also found that the incorporation of TiO_2_ enhanced the excellent response of Ti_3_C_2_/TiO_2_ composites to NO_2_ gas [32]. Shi et al. found that the incorporation of nano TiO_2_/SiO_2_ into epoxy resin enhanced the corrosion resistance using electrochemical impedance spectroscopy (EIS) and salt spray test [33]. This suggests that TiO_2_ may be used as a filler to improve the NO_2_ aging resistance of SIR.

Therefore, in this paper, 2D titanium dioxide nanosheets (TNS)/RTV are prepared and a NO_2_ aging simulation is performed. The electrochemical properties of NO_2_-aged RTV composited samples in an aqueous solution were studied by the EIS technique. Finally, the experimenters systematically explored the influence of TNS on the water permeability of silicone rubber after the NO_2_ aging treatment.

## 2. Materials and Methods

### 2.1. Materials

Titanium dioxide was bought from Degussa Co., Ltd., Shainghai, China. Hydroxyl-terminated polydimethylsiloxane was provided by Guobang Chemical Co., Ltd., Jinan, China. Sodium hydroxide (NaOH) and hydrochloric acid (HCl) were provided by Sinopharm Chemical Reagent Co., Ltd., Beijing, China. Tetraethoxysilane (TEOS) and aminopropyltriethoxysilane (APTES) were purchased from Macklin Biochemical Technology Co., Ltd., Guangzhou, China. Aluminum hydroxide was provided by Aluminum Corporation of China, Zibo, China.

### 2.2. Preparations

Preparation of pure TNS: A total of 0.8 g Titanium dioxide was mixed into 50 mL of a 10 mol/L NaOH solution in a Teflon autoclave, and a hydrothermal process was performed at 130 °C for 3 h. Then, the hydrothermal precipitate obtained by cleaning with distilled water was poured into a 60 mL 0.1 mol/L HCl solution and stirred for 2 h. Finally, the product was cleaned with distilled water and then dried at 60 °C.

Preparation of surface functionalization TNS (f-TNS): First, the commixed silane of APTES and TEOS was prepared according to a mass percentage of 25:75; then, ethanol, water, and commixed silane were blended with a volume percentage of 70:15:15. After completion, TNS was added to the blended solution. The pH of the blended solution dispersed with TNS was adjusted to 4.5 with hydrochloric acid and left at 25 °C for 40 h. After that, the pH of the solution was adjusted to 8.5 with a sodium hydroxide solution (2.5 wt.%) and kept at 65 °C for 2 h. The underlying sediment was separated by centrifugation, cleaned and dried. f-TNS were obtained.

Preparation of nanocomposite coatings with different filler contents (0.1, 0.3, 0.5, 0.7, 1 wt.% f-TNS/RTV): Hydroxyl-terminated polydimethylsiloxane, f-TNS, and aluminum hydroxide were blended at 120 °C for 4 h. When the temperature of the mixture dropped to 40 °C, the other additional fillers were mixed, and this system was blended continuously for 1 h. Then, the mixture was poured over the glass slide and cured at 25 °C for 24 h to form the f-TNS/RTV nanocomposite coating. The resulting coating thickness was 50 ± 2 μm.

NO_2_ corrosion aging treatment: The f-TNS/RTV nanocomposite coating samples were put into a NO_2_ generation device for 24 h and the aged RTV coating samples were obtained. The NO_2_ generation device refers to the previous article [20].

### 2.3. Characterization

The f-TNS nanosheets were characterized by scanning electron microscopy (SEM, Hitachi S-4800, Hitachi, Tokyo, Japan) to obtain morphologies. The chemical environment of surface elements of the TNS was analyzed by X-ray photoelectron spectroscopy (XPS, ESCALAB 250Xi, Thermo Fisher Scientific, Shanghai, China). The EIS test workstation was the CS310 Electrochemical workstation (Coster, Wuhan, China). In addition, all samples were soaked in a 3.5 wt.% NaCl solution during the EIS test [34,35].

## 3. Results and Discussion

**Figure 1:**
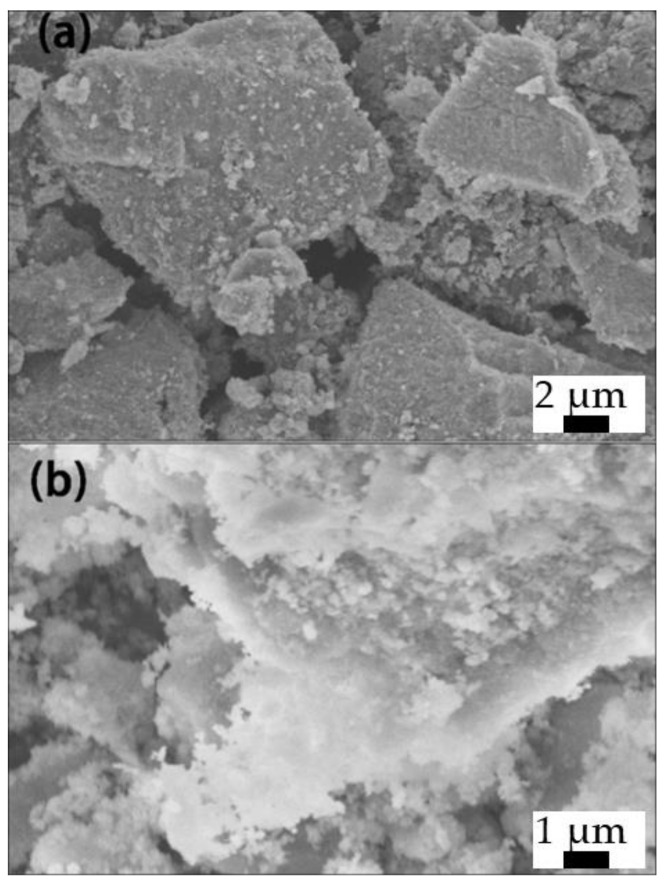
SEM images of pure TNS (**a**) and f-TNS (**b**).

SEM was used to observe the surface morphology of TNS before and after modification. As can be seen in Figure 1a, the TNS presents a big volume agglomeration state because the two-dimensional nanosheets such as TNS have van der Waals forces between them. After modification of TEOS and APTES, particle objects appear on the surface, which may be the condensation of silane and hydroxyl on the surface of TNS to form organosilicon nanoparticles, which can improve the compatibility of inorganic TNS nanosheets in RTV.

**Figure 2:**
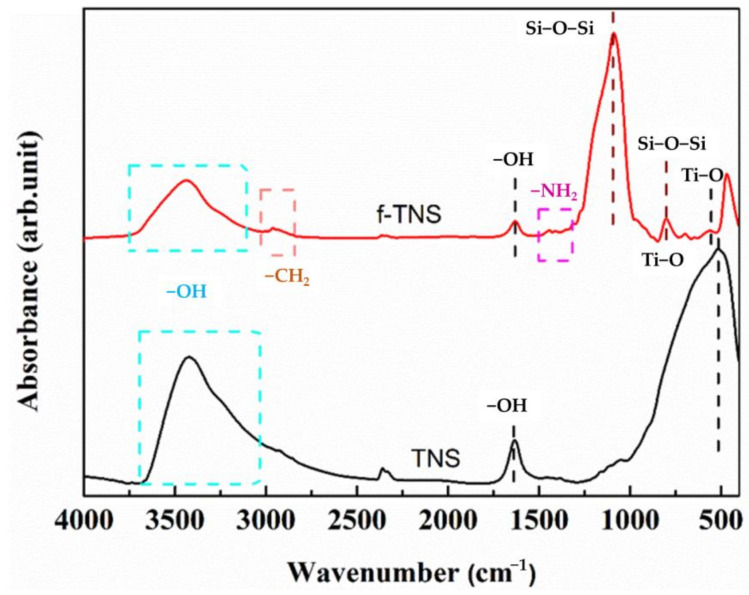
ATR-FTIR spectrogram of TNS and f-TNS.

Figure 2 shows the ATR-FTIR test results of pure TNS and f-TNS nanosheets. TNS and f-TNS nanosheets had the same peaks in FTIR spectra, including −OH at 3200 and 1650 cm^−1^_,_ and Ti−O−Ti at 510 cm^−1^. The presence of surface hydroxyl groups in TNS provided an active site for graft condensation for silane modification. After modification by TEOS and APTES [35], the peak of Ti−O−Ti in f-TNS was offset and moved to 530 cm^−1^, indicating that the hydroxyl group on TNS had a condensation reaction with a silane coupling agent, thus making the peak of the Ti−O−Ti shift. The appearance of Ti−O−Ti peaks indicates that the modification of TEOS and APTES does not affect the structure of TNS, which still maintains its chemical structure. In addition, four new vibration peaks appeared at 2960, 1550, 1100, and 810 cm^−1^, which were, respectively, attributed to −CH_2_, −NH_2_, Ti−O−Si, and asymmetric stretching vibration Si−O−Si [36,37,38]. According to the SEM results of the above section, the surface of TNS was coated with some fluffy nano-silicone particles modified by organosilicon. Therefore, the shift of the Ti−O−Ti peak and the appearance of methylene, amino, Ti−O−Si, and Si−O−Si once again prove that silane was successfully grafted onto the surface of TNS.

**Figure 3:**
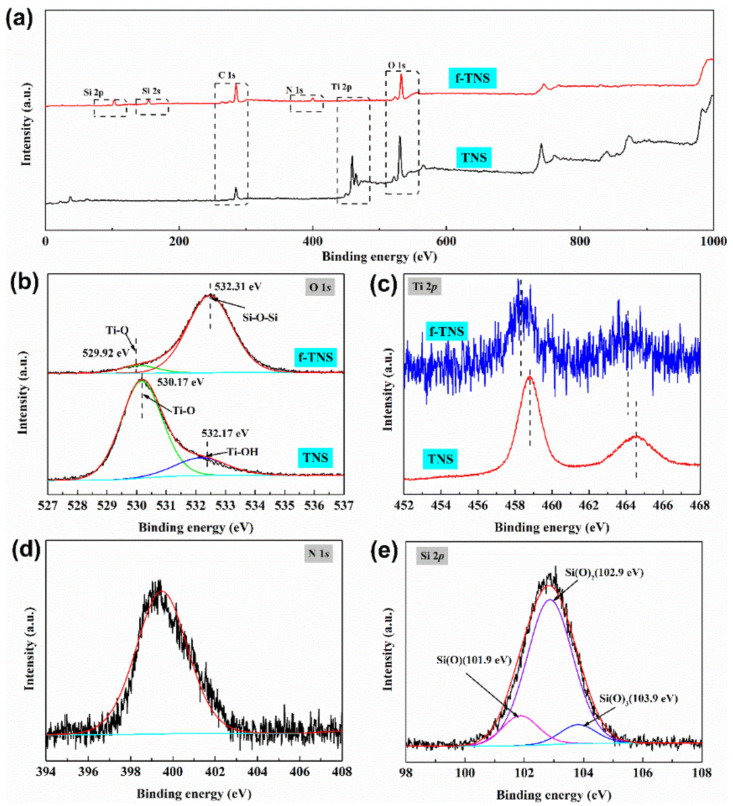
XPS full spectra analysis of pure TNS and f-TNS (**a**), XPS high-resolution spectra of O 1s (**b**), Ti 2p (**c**), N 1s (**d**), and Si 2p (**e**).

**Figure 4:**
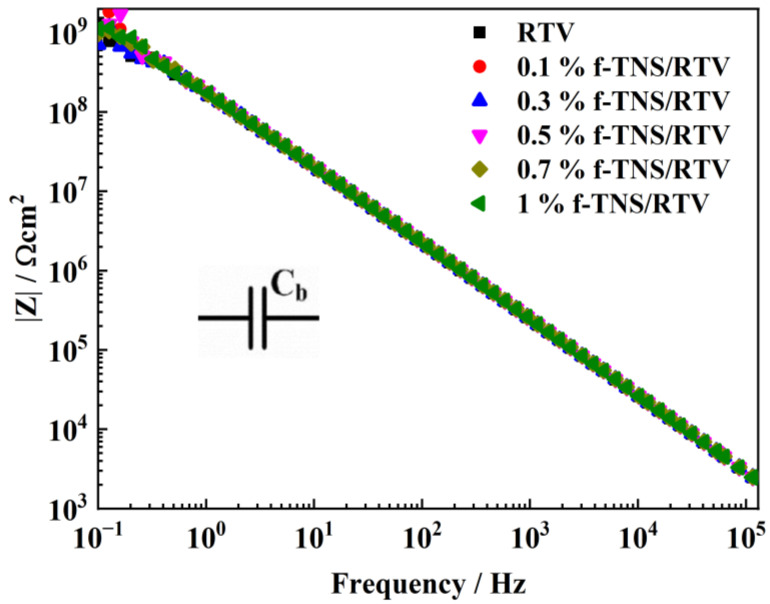
The electrochemical impedance modulus at a 0.1 Hz |Z| cave of unaged RTV with series of f-TNS composite proportions after soaking following a 96 h immersion in a NaCl solution.

To further determine the surface chemical state of modified TNS nanosheets, XPS was used to analyze the pure TNS and f-TNS. As can be seen in Table 1, Ti and O (18.58:46.91 at %) are the main element composition of TNS, and the presence of carbon is caused by the influence of the substrate during the test. For f-TNS nanosheets, the elemental composition is C, N, O, Ti, and Si (49.11:1.25:27.19:4.58:17.86 at %). The additional peak of Si and N indicates that silane was modified on the surface of the sheet. The bonding states of Ti 2p, N 1s, O 1s, and Si 2p of the f-TNS nanosheet and TNS are distinguished by a suitable combination of Lorentz and Gaussian functions. In Figure 3b, the O 1s XPS spectral peaks of the original TNS showed two peaks at 530.17 eV and 532.17 eV, corresponding to Ti−O and Ti−OH [39]. The Ti−O bond of the modified TNS appeared at 529.92 eV and was skewed towards the lower binding energy. The Ti−O movement indicated that the Ti−O−Si bond was formed on the surface of TNS. It was speculated that the hydrogen in Ti−O−H was partially replaced by silicon in Ti−O−Si, because Si is less electronegative than H, so the electron density in O is higher. As shown in Figure 4c, after modification, the characteristic peak of Ti 2p also deviated towards low binding energy, again indicating the formation of the Ti−O−Si bond. It shows that the combination of TNS and silane is not a simple physical mixing. Figure 4d shows three typical stretching vibration peaks of Si 2p at 101.9, 102.9 and 103.9 eV in Si(−O)_1_, Si(−O)_2_ and Si(−O)_3_ condensation systems [40]. The presence of Si−O−Si and Si−O−Ti indicated that some silane was successfully grafted onto the surface of the nanosheets according to the covalent bond.

The EIS of an unaged RTV coating with a series of f-TNS composite proportions is shown in Figure 4. After a 96 h immersion in a NaCl solution, the electrochemical impedance modulus at 0.1 Hz (|Z|) of RTV coating remained at 10^9^ Ω cm^2^, indicating that the introduction of f-TNS did not destroy the water resistance of the coating, which was regarded as a pure capacitor [34], even though the coating maintained good integrity after a 96 h immersion.

**Figure 5:**
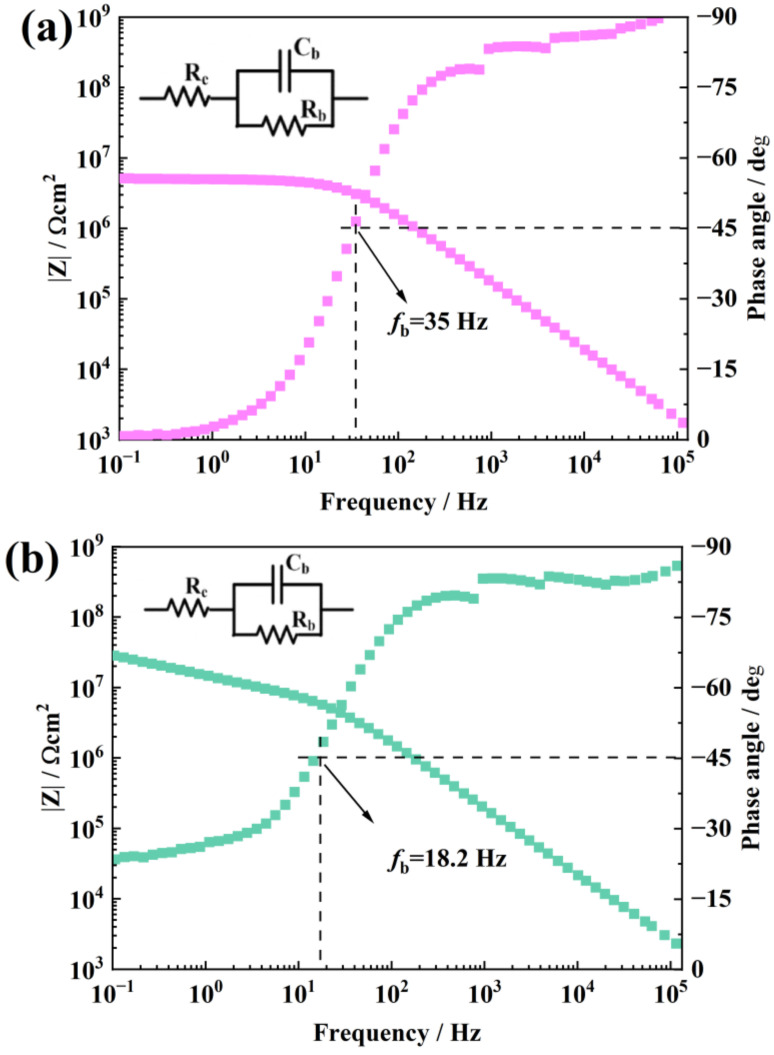
The EIS Bode plots of pure RTV sample after NO_2_ corrosion (**a**) and a 0.1 wt.% f-TNS/RTV sample after NO_2_ corrosion (**b**).

Figure 5 is the EIS of pure RTV and a 0.1 wt.% f-TNS/RTV after NO_2_ aging for 24 h. The water permeability of aged pure RTV coating decreases, obviously. When the coating was immersed in a 3.5 wt.% NaCl solution for 96 h, the |Z| decreased obviously compared with the unaged coating. |Z| decreased from 10^9^ Ω cm^2^ to about 8.2 × 10^6^ Ω cm^2^, which shows that due to NO_2_ corrosion, the water permeability of RTV coating was destroyed. In addition, the EIS frequency at 45° is called the breakpoint frequency $f_{b}$, and its magnitude could qualitatively analyze the delamination of the coating. The bigger the $f_{b}$, the more severe the coating delaminate [34,35]. After NO_2_ aging, the $f_{b}$ of pure RTV was about 35 Hz, which implies that aging causes a certain degree of delamination. As shown in Figure 5b, when a 0.1 wt.% f-TNS nanosheet was filled, the |Z| of the sample increased compared with that of pure RTV, about 1.24 × 10^7^ Ω cm^2^, and the $f_{b}$ of the sample decreased to 18.2 Hz. This shows that the incorporation of the nanosheets slows down the diffusion of NO_2_ gas inside the coating to a certain extent and improves water permeability. However, there is still a relatively serious delamination of the coating. The experiment continues to increase the filling amount of TNS to explore the coating formulation with the best water permeability effect.

**Figure 6:**
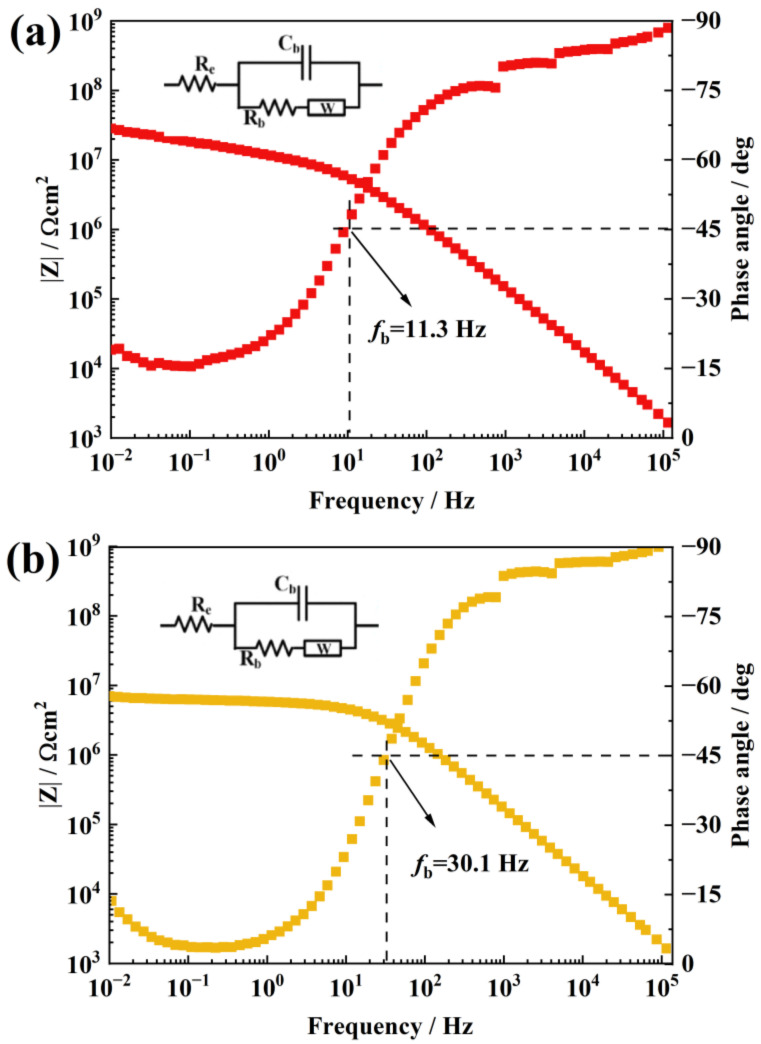
The EIS Bode plots of a 0.3 wt.% f-TNS/RTV sample after NO_2_ corrosion (**a**) and a 0.5 wt.% f-TNS/RTV sample after NO_2_ corrosion (**b**).

Figure 6 is the EIS of the aging 0.3 and 0.5 wt.% f-TNS/RTV. The |Z| of a 0.3 wt.% f-TNS/RTV is about 1.82 × 10^7^ Ω cm^2^, and the $f_{b}$ also obviously decreases, which is about 11.3 Hz. Only one time constant was observed in the Byrd diagram, and a capacitive phenomenon was observed in a wide frequency range (10^2^–10^5^ Hz), indicating that the increase in the functionalized nanosheet content f-TNS can largely delay and prevent the diffusion of NO_2_ gas inside the RTV sample. This extension of the gas diffusion path could protect internal RTV from NO_2_ corrosion so that the coating can maintain better integrity. As can be seen from Figure 6b, for a 0.5 wt.% f-TNs/RTV, the |Z| of the coating decreases again, about 6.28 × 10^6^ Ω cm^2^, compared with that of the coating with a 0.3 wt.% filling. Accordingly, $f_{b}$ is also increased to 30.1 Hz. This indicates that a 0.5 wt.% f-TNs/RTV has a lesser NO_2_ blocking function than a 0.3 wt.% f-TNs/RTV, although the water permeability is better than that of pure RTV. It is speculated that the increase in f-TNS content may cause the aggregation of f-TNS in RTV, and the defects caused by agglomeration may become the aging breakthrough channel of NO_2_, and eventually become the decline of water resistance.

**Figure 7:**
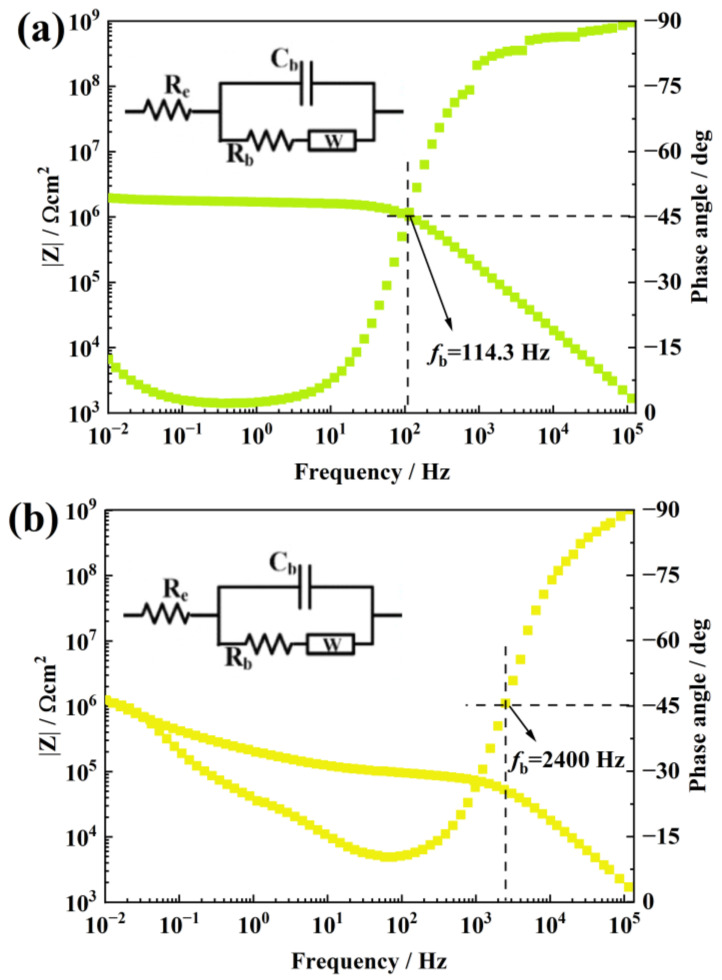
The EIS Bode plots of a 0.7 wt.% f-TNS/RTV sample after NO_2_ corrosion (**a**) and a 1.0 wt.% f-TNS/RTV sample after NO_2_ corrosion (**b**).

Figure 7 is the EIS of aged 0.7 wt.% and 1.0 wt.% f-TNS/RTV. Compared with the above coating, the |Z| of the RTV is further reduced to about 1.78 × 10^6^ Ω cm^2^. In addition, the $f_{b}$ also become bigger once more, about 114.3 Hz. It implies that the sample is severely stratified. The |Z| of a 1.0 wt.% f-TNS/RTV shows a more serious decrease, dropping to 4.2 × 10^5^ Ω cm^2^, which is smaller than that of the pure RTV coating. Meanwhile, its $f_{b}$ rises suddenly to 2400 Hz, which is 212.4 times the breakpoint frequency of a 0.3 wt.% f-TNS/RTV. These EIS results show that the excessive addition of the filler greatly reduces the resistance of the coating to NO_2_. We guess this may be due to the size of the nanometer sheet is so small that it is easy to reunite and form a large NO_2_ seepage channel. The filler agglomeration makes the NO_2_ in the coating internally spread more easily. The damage of NO_2_ to the coating microstructure is more serious, which affects the integrity of the coating, making its water barrier ability greatly reduced.

**Figure 8:**
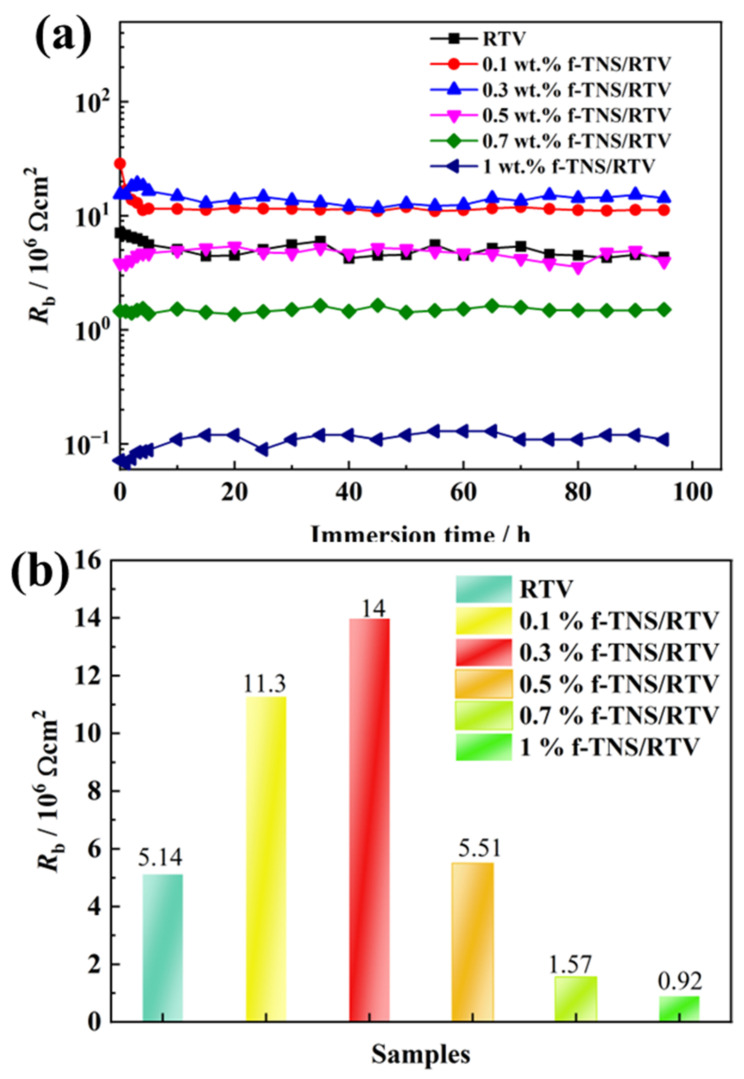
The resistance change cave of an aged RTV sample with a series of f-TNS composite proportions at different soaking times (**a**), the resistance of an aged RTV sample with a series of f-TNS composite proportions after soaking for 96 h (**b**).

Figure 8 shows the variation curve of fitting RTV coating resistance (R_b_) with a series of f-TNS composite proportions at different soaking times. Taking the R_b_ curve of pure RTV as a reference, the coating R_b_ increases when a 0.1 wt.% f-TNs is filled, about 1.13 × 10^7^ Ω cm^2^. These results show that the composite proportion of f-TNS can play a certain role in blocking NO_2_ gas. For a 0.3 wt.% f-TNs/RTV, the coating R_b_ continues to increase to about 1.4 × 10^7^ Ω cm^2^, which is the highest value for the whole set of samples. As the TNS content continues to increase, the coating R_b_ begins to decrease. Especially when the TNS content increases to 1 wt.%, its R_b_ is about five times lower than pure RTV, about 9.2 × 10^5^ Ω cm^2^. The overall result is that with the increase in f-TNS content, the coating R_b_ first increases and then decreases; that is, the best filling ratio of f-TNS is about 0.3 wt.%.

**Figure 9:**
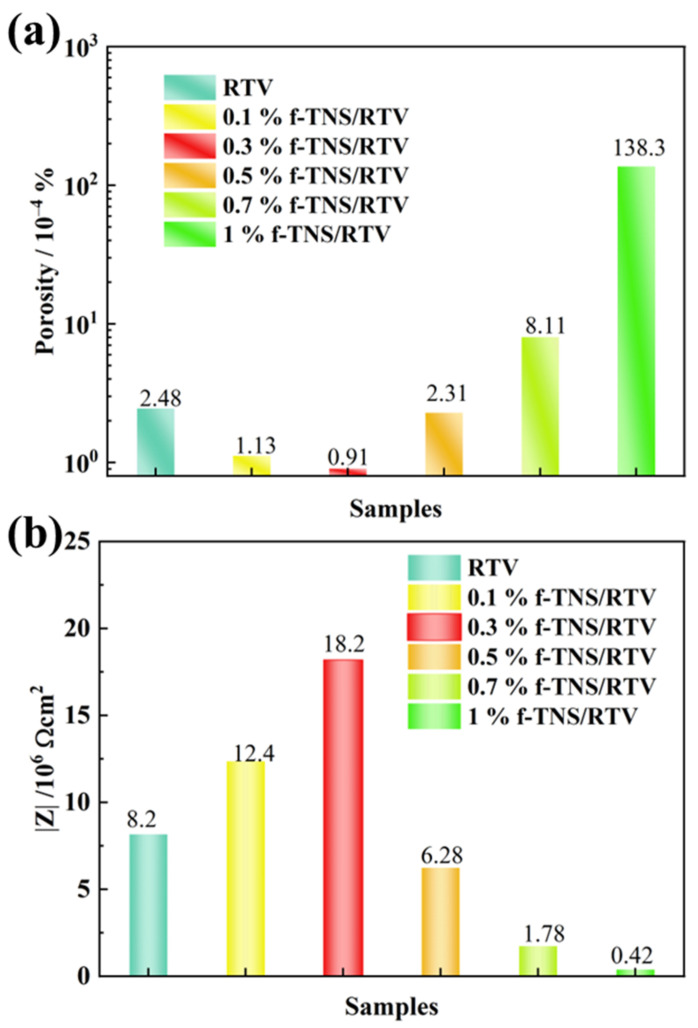
The bar graph of the porosity (**a**) and the |Z| (**b**) of the RTV coatings with a series of f-TNS composite proportions after NO_2_ corrosion aging.

Figure 9 shows the variation curves of the porosity and the |Z| of RTV samples with a series of f-TNS composite proportions corroded by NO_2_. The calculation method of porosity is referred to in the previous article [20]. It can be seen from Figure 9a that with the increase in f-TNS, the porosity of the coating decreases first and then increases. The 0.3 wt.% f-TNS/RTV has a minimum porosity of about 0.91 × 10^−4^%. The porosity of pure RTV coating after NO_2_ corrosion aging is 2.73 times that of f-TNS/RTV filled with 0.3 wt.%. When the composite proportions of f-TNS nanosheets reached 1 wt.%, the porosity of the aged RTV sample boomed by more than 100 times, reaching 1.38 × 10^−2^%. It can be seen from Figure 9b that with the increase in f-TNs content, the |Z| of the coating increases first and then decreases. Especially for a 0.3 wt.% f-TNS/RTV, the |Z| reaches 1.82 × 10^7^ Ω cm^2^, which is 2.23 times higher than the 8.2 × 10^6^ Ω cm^2^ of pure RTV. As the f-TNS continues to increase to 1 wt.%, the |Z| of the RTV sample decreases to a minimum of about 4.2 × 10^5^ Ω cm^2^, which is about 50 times smaller than that of the pure RTV sample. The results of the porosity and |Z| also indicate that the optimal f-TNs composite proportion is about 0.3 wt.%.

**Figure 10:**
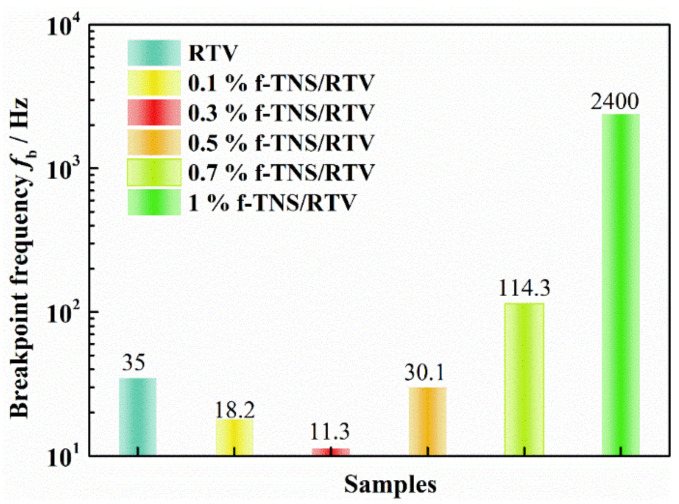
The bar graph of the breakpoint frequency $f_{b}$ of RTV coatings with a series of f-TNS composite proportions after NO_2_ corrosion aging.

In Figure 10, the influence of f-TNS composite proportions on the delamination of RTV can be seen more directly from the change curve of $f_{b}$. With the increase in the composite proportion of f-TNS, the $f_{b}$ of the RTV sample first decreases and then increases. Especially when the composite proportion of f-TNs is 0.3 wt.%, the $f_{b}$ of the RTV sample is reduced to 11.3 Hz. And it is much smaller than that of pure RTV, which is 35 Hz. However, when the composite proportions of f-TNS in RTV increase to 1 wt.%, the $f_{b}$ of the RTV coating increases to 2400 Hz, which is 212.4 times that of filling 0.3 wt.%.

Since RTV coating samples with a series of f-TNS composite proportions have excellent water resistance performance before NO_2_ aging, it indicates that the introduction of f-TNS does not destroy the water resistance of the coating. The data of R_b_, |Z|, porosity and $f_{b}$ of the aged RTV coatings with a series of f-TNS composite proportions show that the introduction of f-TNS can improve the NO_2_ corrosion aging resistance and water permeability of aged RTV. The composite coating has the best performance when the composite proportion is 0.3 wt.%, which may be because a small amount of f-TNS has a good dispersion in the coating, which can play a role of a physical barrier and increase the diffusion path, delaying the corrosion of NO_2_ on the coating. However, with the continuous increase in nanosheet content, f-TNS is easy to agglomerate, and the defects caused by agglomeration become a breakthrough for NO_2_ corrosion, and the aging is more serious than that of pure RTV, resulting in a more significant rise in the water permeability of the RTV coating.

## 4. Conclusions

In this paper, the composition and chemical structure of f-TNS and the influence of f-TNS on the water permeability of RTV after NO_2_ aging treatment were studied. SEM, FTIR, and XPS jointly confirmed that silane was successfully grafted onto TNS and the f-TNS was formed. The grafted silane enhanced the interfacial compatibility of 2D sheet TNS in RTV. After NO_2_ aging for 24 h and soaking in sodium chloride solution for 96 h, the |Z| and R_b_ of the f-TNS/RTV sample both first increased and then decreased, whereas the porosity and the $f_{b}$ both first decreased and then increased. All of the indexes reached the optimal value when the composite proportion of f-TNS was 0.3 wt.%, which confirms that the aged 0.3 wt.% f-TNS/RTV has the best water permeability. In short, an about 0.3 wt.% f-TNS composite proportion has good dispersion in the RTV coating, which can play the role of the physical barrier and increase the diffusion path, delaying the corrosion of NO_2_ on the coating. However, with the continuous increase in nanosheet content, f-TNS is easy to agglomerate, the defects caused by agglomeration become a breakthrough for NO_2_ corrosion, and the aging is more serious than that of pure RTV, resulting in a more significant decline in the water barrier property of the coating. This study investigated a possible method to improve the NO_2_ aging resistance of RTV, which has the minimum water permeability and further provides the safe operation of power grid insulators.

## Figures and Tables

**Table 1:** The atomic proportion of each element of TNS and f-TNS.

	C/at %	N/at %	O/at %	Ti/at %	Si/at %
TNS	34.51	/	46.91	18.58	/
f-TNS	49.11	4.25	27.19	1.58	17.86

## Data Availability

Raw data are available upon request.
